# Effect of shrouding CH_4_ flow rate on flow field and stirring ability of coherent jet in steelmaking process

**DOI:** 10.1186/s40064-016-3212-3

**Published:** 2016-09-20

**Authors:** Fuhai Liu, Dongbai Sun, Rong Zhu, Rongfang Su, Xueyi Wang

**Affiliations:** 1 National Center for Materials Service Safety, University of Science and Technology Beijing, Beijing, 100083 China; 2 Beijing Key Laboratory of Research Center of Special Melting and Preparation of High-end Metal Materials, University of Science and Technology Beijing, Beijing, 100083 China; 3 Rongcheng Jingye Technology Limited Company Beijing, Beijing, 100083 China; 4 Tianjin Pipe Co. Ltd., Tianjin, 300301 China

**Keywords:** Electric arc furnace, Coherent jet, Combustion experiment, Numerical simulation

## Abstract

Characteristics of flow field and stirring ability of coherent jet with various shrouding CH_4_ flow rates on the molten bath were studied by combustion experiment and numerical simulation. The axial velocity and total temperature distributions of coherent jet under hot (1700 K) and cold (298 K) ambient condition were analyzed. The Eddy Dissipation Concept model was used in simulation with detail chemical kinetic mechanisms, and the numerical simulation results were agreed well with the combustion experiment in this research. Based on the simulation and experiment results, when the CH_4_ rate was 230, 207 and 184 Nm^3^/h, their disparity rate of average velocity and total temperature was small than 5 and 6 %, respectively, at high ambient temperature. Hence, the same stirring effect might be achieved by those three kinds of CH_4_ flow rates in EAF steelmaking process. According to the industrial application research, the best CH_4_ flow rate is 184 Nm^3^/h, which could stir molten bath well and reduce energy consumption in steelmaking process.

## Background

During the steelmaking process, the main equipment to supply oxygen into the molten bath is oxygen lance, which is used widely in the basic oxygen furnace (BOF) and the electric arc furnace (EAF). Moreover, the oxygen lance also have a key function in dephosphorization and stirring the molten pool. In order to achieve a great stirring ability and high reaction rate, the Laval nozzle is adopted to increase the velocity of oxygen jet (Deo and Boom 1993; Naito et al. 2000). In the Laval nozzle, the high pressure energy of oxygen jet is transformed into its kinetic energy, and the jet would be accelerated to 2.0 Mach number. After that, the velocity of oxygen jet begins to reduce due to entrainment of ambient gas, which forms potential core, supersonic core and subsonic zone. With increasing distance from the tip of nozzle, the impact force of the oxygen jet decreases which would reduce the mass transfer processes and the reaction rates in the furnace (Tago and Higuchi 2003; Wang et al. 2010).

To solve that problem, the coherent jet technology has been applied to electric arc furnace steelmaking process (Liu et al. 2005; Mathur 1999a), which could prolong the length of potential core and increase the kinetic energy of the main oxygen jet. The key of this technology is suppressing the ambient gas to interact with main oxygen jet by a shrouding flame. Therefore, the oxygen jet could keep original diameter and velocity over long distance, and remain the stirring ability. Moreover, with the greater penetration capacity, the supersonic oxygen jet could deliver greater amounts of oxygen into the molten bath and decrease the splash of liquid slag, comparing with the traditional supersonic oxygen lance (Nordquist et al. (2006); Mahoney (2008)).

The coherent jet technology was proposed by Mathur and Anderson et al. (1998), and then Mathur (1999b) showed the fundamental results in industrial production. Sarma et al. (1998) analyzed the characteristics of supersonic jet and coherent jet at different ambient temperatures by combustion experiment. Alam et al. (2010) reported the behaviors of the supersonic jet with and without shrouding flame by the numerical simulation and validated their results against previously experimental data. To date, although substantial works have been performed for researching the differences between conventional oxygen jet and coherent jet (Anderson et al. 1998; Mathur 1999b; Sarma et al. 1998; Alam et al. 2010a; Mahoney 2010; Meidani et al. 2004; Liu et al. 2016; Jeong et al. 2004), little research has been paid to how the flow field is influenced by flow rate of shrouding flow.

The present study is to make some contributions to address this deficit by combustion experiment and numerical simulation. During the combustion process, the process products are formed such as CH_5_, CH_2_ and OH, which could not be tracked by conventional one-step complete combustion. Therefore, the numerical simulation is performed with detail chemical kinetic mechanisms to analyze the effect of process products on coherent jet flow field, and EDC model is used to analyze how the shrouding flow rate affects the potential core length and stirring ability of coherent jet under two kinds of ambient temperatures. Based on results, the metallurgical effects of various CH_4_ flow rates are analyzed in a 75 t electric arc furnace.

## Combustion experiment

**Fig. 1:**
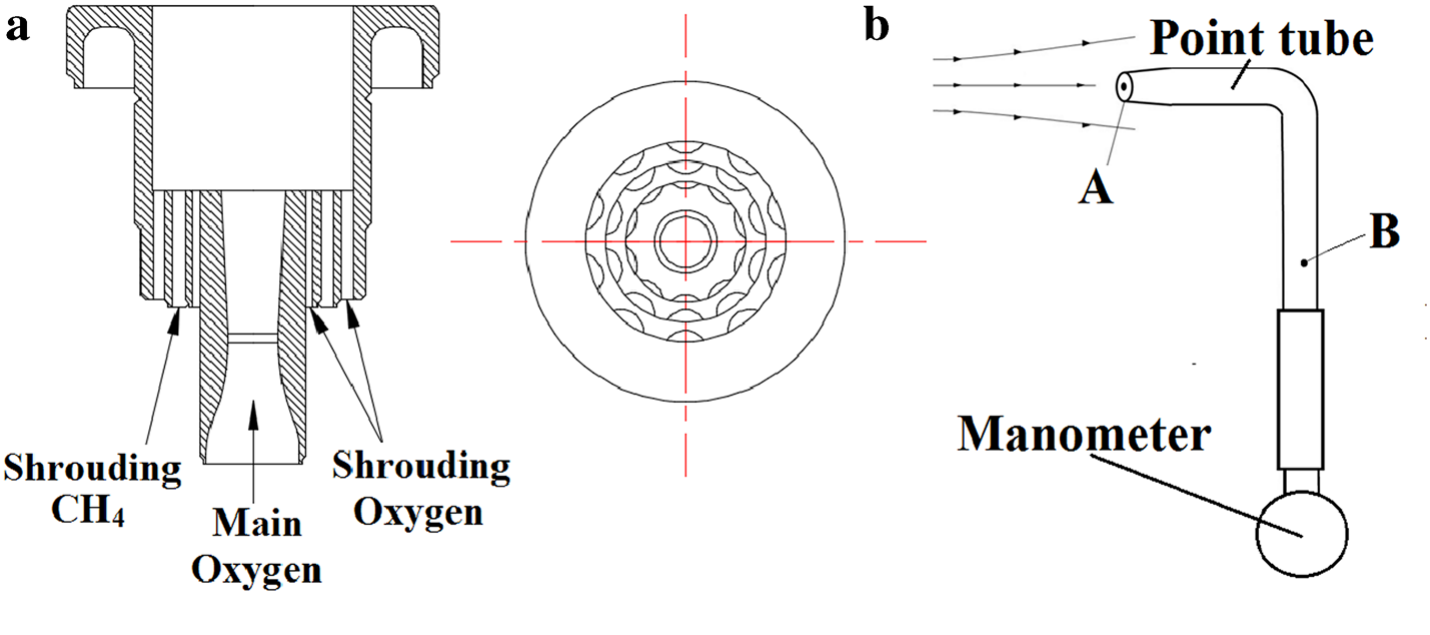
**a** Cross sectional and front view of supersonic coherent jet nozzle. **b** The schematic diagram of point tube

The present research used a combustion system to produce a hot ambient temperature for the coherent jet. The details of the system were described in Ref. (Mardani et al. 2010), and only a brief description was given here. In this paper, the coherent jet lance had a water-cooled system, and the design flow rate of main oxygen was 2300 Nm^3^/h. Moreover, the throat and exit diameter of Laval nozzle was 25.3 and 32.9 mm, respectively. There were three concentric rings supplying the shrouding CH_4_ and O_2_, as presented in Fig. 1a. The inner hydraulic, intermediate and outer hydraulic diameter is 3.2, 4.5 and 4.8 mm, respectively. For combustion experiment and numerical simulation, the same parameters of supersonic jet nozzle were applied in this paper.

The point tube with water-cooling was adopted to measure the static pressure and total pressure of coherent jet in the combustion experiment, as shown in the Fig. 1b. The Mach number of coherent jet should be calculated by following formation (Anderson 2013):

1 $$Ma^{2} = \frac{2}{\gamma - 1}\left[ {\left( {\frac{{p_{0} }}{p}} \right)^{(\gamma - 1)/\gamma } - 1} \right]$$

where P_0_ is the pressure at the A location, P is the pressure at the B location and γ is the ratio of heat capacity.

## Numerical model

### Governing equations

The numerical simulations were conducted by integrating the Navier–Stokes equations with Reynolds averaging method, and following equations was used to simulated combustion process (Malalasekera and Versteeg 2007).

Mass conservation equation

2 $$\frac{\partial \rho }{\partial \tau } + \frac{{\partial \rho U_{i} }}{{\partial X_{i} }} = 0$$

Momentum conservation equation

3 $$\frac{\partial }{\partial \tau }(\rho \overrightarrow {v} ) + \nabla \cdot (\rho \overrightarrow {v} \overrightarrow {v} ) = - \nabla P + \nabla \left[ {\mu_{e} \left( {\nabla \overrightarrow {v} + \nabla \overrightarrow {v}^{T} } \right)} \right] + \rho \overrightarrow {g} + f_{\alpha }$$

Energy equation

4 $$\frac{\partial }{\partial \tau }(\rho E) + \nabla \cdot (\overrightarrow {v} (\rho E + \rho )) = \nabla (k_{eff} \nabla T - \sum\nolimits_{j} {h_{i} } \overrightarrow {{J_{j} }} + (\overrightarrow {{\tau_{eff} }} \overrightarrow {v} )) + S_{h}$$

In (2–4), $\overrightarrow {v}$ was the velocity vector; k_eff_ was the effective conductivity and $\overrightarrow {{J_{j} }}$ was the diffusion flux of species j; S_h_ included the heat of chemical reaction, and any other volumetric heat sources.

The discrete ordinate (DO) radiation model (Christo and Dally 2005; Chui and Raith 1993) with weighted sum of gray gas model (WSGGM) was used to model the radiation of the combustion process. In the present study, the modified k–ε model with the standard wall function was implemented for modeling the turbulent flows. The standard k–ε model (Launder and Spalding 1972) was a semi-empirical model based on model transport equations for the turbulence kinetic energy (k) and its dissipation rate (ε).

Turbulence kinetic energy equation for EDC model (k equation):

5 $$\frac{\partial (\rho k)}{\partial \tau } + \frac{\partial }{{\partial x_{i} }}(\rho ku_{i} ) = \frac{\partial }{{\partial x_{j} }}\left[ {\left( {\mu + \frac{{\mu_{t} }}{{\sigma_{\kappa } }}} \right)\frac{\partial k}{{\partial x_{j} }}} \right] + G_{k} + G_{b} - \rho_{\varepsilon } - Y_{M} + S_{k}$$

Turbulence dissipation equation for EDC model (ε equation):

6 $$\frac{\partial (\rho \varepsilon )}{\partial \tau } + \frac{\partial }{{\partial x_{i} }}(\rho \varepsilon u_{i} ) = \frac{\partial }{{\partial x_{j} }}\left[ {\left( {\mu + \frac{{\mu_{t} }}{{\sigma_{\varepsilon } }}} \right)\frac{\partial \varepsilon }{{\partial x_{j} }}} \right] + C_{1\varepsilon } \frac{\varepsilon }{\kappa }(G_{k} + C_{3\varepsilon } G_{b} ) - C_{2\varepsilon } \rho \frac{{\varepsilon^{2} }}{\kappa } + S_{\varepsilon }$$

In the equations, the G_k_ and G_b_ presented was the generation of turbulence kinetic energy due to the mean velocity gradient and the buoyancy, respectively. Y_M_ represented the contribution of the fluctuating dilatation in compressible turbulence to the overall dissipation rate. S_k_ and Sε were user-defined source terms. In combustion process, the temperature gradient was an important factor to obtain the flow field (Mardani et al. 2010; Magnussen and Hjertager 1977). Hence, the turbulent viscosity (μ_t_) is addressed as the following equation (Alam et al. 2010b):

7 $$\mu_{t} = \frac{{0.09\rho \varepsilon^{2} }}{{\left[ {1 + \frac{{1.2T_{g}^{0.6} }}{{1 + f({\text{M}}_{\tau } )}}} \right]k}}$$

The EDC model with overall and detailed chemical kinetic mechanisms (GRI-Mech 3.0) were presently used for the modeling of reactions (Frassoldati et al. 2010; Mardani and Tabejama 2010; Galletti et al. 2009). The GRI-Mech 3.0 was the full chemical mechanism, which consisted of 53 species and 325 reversible reactions. In EDC model, the species conservation equation for chemical species taken the following general form:

8 $$\frac{\partial }{\partial t}(\rho Y_{i} ) + \nabla \cdot (\rho \overrightarrow {v} Y_{i} ) = - \nabla \overrightarrow {{J_{i} }} + R_{i}$$

where Y_i_ was the local mass fraction of each species (i), $\overrightarrow {{J_{i} }}$ was the diffusion flux of species, and R_i_ was the net rate of production of species by chemical reaction. The length fraction of the fine scales (ξ) and the residence chemical time scale (τ) of fluid in the fine structures was expressed by:

9 $$\xi = C_{\xi } \left( {\frac{v\varepsilon }{{k^{2} }}} \right)^{2} ,\quad \tau = C_{\tau } \left( {\frac{v}{\varepsilon }} \right)^{{\frac{1}{2}}}$$

where C_ξ_ and C_s_ were time scale constants equal to 2.138 and 0.408, respectively.

### Simulation details

**Fig. 2:**
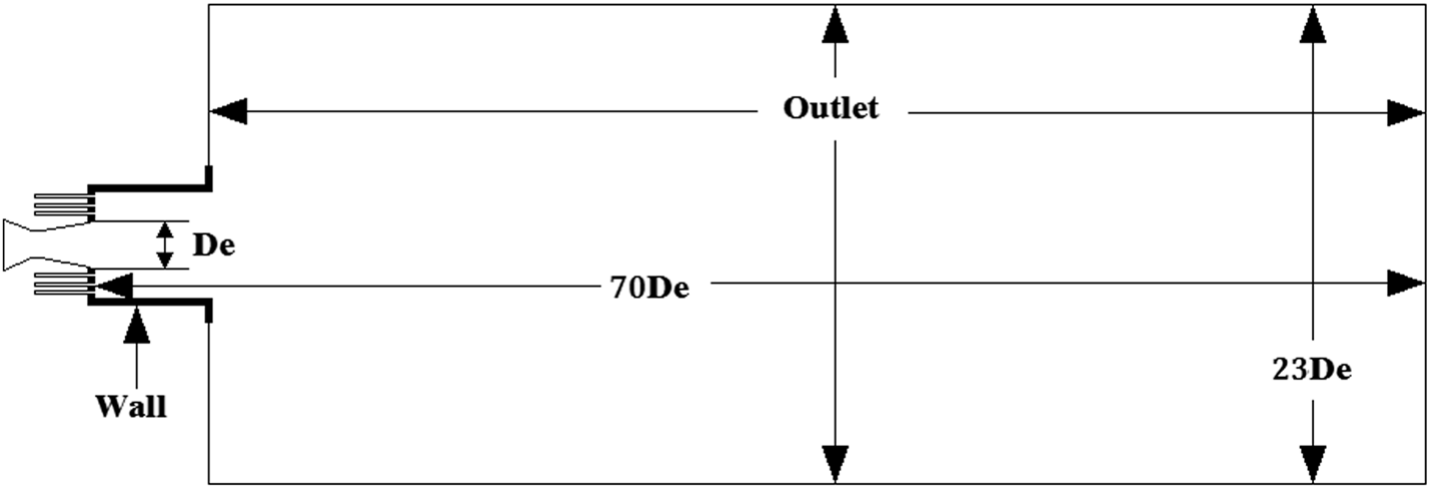
Boundary conditions of the computational domain

Because the vortices in flow field could not be correctly reflected by the symmetry of the system, a 3D geometrical model of computation was constructed in Fig. 2. The computational domain started at the entrance of the coherent jet nozzle, and extended 70D_e_ downstream in the axial direction and 23D_e_ in the radial direction.

The main oxygen used pressure inlet condition, surrounding CH_4_ and O_2_ adopted mass-flow inlet condition for and outlet position of combustion zone adopted pressure-outlet. The Table 1 listed the detail values of boundary conditions.

**Table 1:** Specifications of boundary conditions

Name of boundary	Type of boundary conditions	Values
Main oxygen inlet	Stagnation pressure (Pa)	768,545
	Mach number	2.0
	Mass fractions (wt%)	O_2_ = 100
	Oxygen temperature (K)	298
CH_4_ inlet	Mass flow rate (kg/s)	0.0456/0.0411/0.0365/0.0319/0.0274/0.0228/0
	Mass fractions (wt%)	CH_4_ = 100
	CH_4_ temperature (K)	298
Surrounding oxygen inlet	Mass flow rate (kg/s)	0.0913/0.0821/0.0730/0.0639/0.0548/0.0456/0
	Mass fractions (wt%)	O_2_ = 100
	Surrounding oxygen temperature (K)	298
Outlet	Static pressure (Pa)	101,325
	Mass fractions (wt%)	O_2_ = 21, N_2_ = 79
Ambient temperature	Initial temperature (K)	298/1700

In present study, The SIMPLE algorithm method (Chui and Raith 1993; Launder and Spalding 1972) was utilized to solve pressure velocity coupling. The second-order upwind scheme was employed for discretizing the equations in order to improve the accuracy of the simulations. Solution convergence was determined by two criteria. The first one was to ensure that the numerical residuals were <10^−6^ for the energy and <10^−5^ for all the other variables. The second criterion was to ensure that the variations between consecutive iterations of temperature and velocity were kept within 10 K and 2 m/s, respectively, at the downstream outlet of the computational domain.

There were large quantities of previous studies (Mardani et al. 2010; Magnussen and Hjertager 1977; Frassoldati et al. 2010; Mardani and Tabejama 2010; Galletti et al. 2009) had used GRI-Mech 3.0 to model the combustion process. Thus, the EDC model with the detailed chemical kinetic mechanism of GRI-Mech 3.0 was used for the modeling of reactions. To reduce the calculation time, the in situ adaptive tabulation (ISAT) model of Pope (1997) was adopted.

**Fig. 3:**
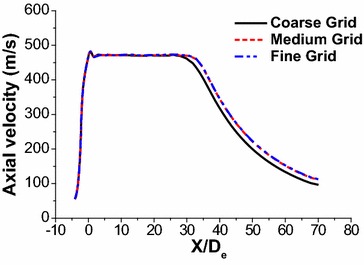
Axial velocity of coherent jet with three grid levels

Figure 3 shows the axial velocity profiles for the simulation results in three grid levels, coarse grid (275,000 cells), medium grid (417,000 cells), and fine grid (564,000 cells), respectively. It seems that the simulation results agree well with the each other, for the medium and the fine grid, and the variation of axial velocity is within 0.7 pct. This result suggests that the solution is not sensitive to the grid. However, the variation calculated with the coarse and medium grid level was about 2.4 pct. Therefore, the mesh of 417,000 cells was used in combustion simulation in consideration of reducing the computational time.

## Results and discussions

### Axial velocity distribution research

**Fig. 4:**
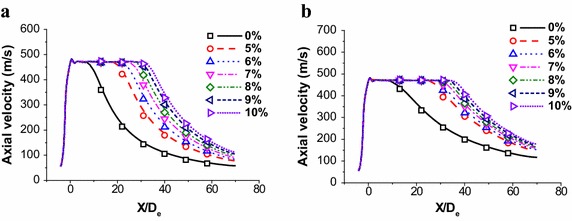
Axial velocity distributions of supersonic jet at the jet centerline. **a** The coherent jet under room ambient temperature. **b** The coherent jet under high ambient temperature

Figure 4 shows the axial velocity distribution of the supersonic oxygen jet with different shrouding CH_4_ flow rates and ambient temperatures. Moreover, the simulation results would be addressed as line or line segment, and the experiment data would be presented as quadrangle, roundness or triangle in Figs. 4 and 6. The mass flow rates of CH_4_ in different unit are shown in the Table 2. In this paper, the percent of main oxygen design flow rate will be used to replace CH_4_ mass flow rate, hereafter. For instance, 10 %-CH_4_ represents the shrouding CH_4_ mass flow rate being 0.0456 kg/s.

**Table 2:** The mass flow rates of CH_4_ in different unit

Mass flow rate of CH_4_ (kg/s)	Pneumatic volume flow rate of CH_4_ (Nm^3^/h)	Pneumatic volume flow rate of O_2_ (Nm^3^/h)	The percent of main oxygen flow rate (%)
0.0456	230	2300	10
0.0411	207		9
0.0365	184		8
0.0319	161		7
0.0274	138		6
0.0228	115		5
0	0		0

Based on average values of different kinds of working conditions in Fig. 4, within 5 nozzle exit diameters from the nozzle exit, all the velocities of main oxygen jets are 471 ± 10 m/s at the jet centerline. As the results show, more shrouding CH_4_ rate does not increase the maximum velocity of main oxygen jet, and it just makes the maximum velocity remain a longer distance at the jet centerline. Therefore, CH_4_ flow rate could only protect the stirring ability of main oxygen jet, but not improve the stirring ability at a certain extent.

1 nozzle exit diameter will be addressed as 1D_e_ hereafter. The potential core of coherent jet is supposed to be end, when the axial velocity of supersonic oxygen jet continues to decline more than 5D_e_ at the jet centerline, and the first point of this kind of downtrend is addressed as the end point of potential core. The length between the end point of potential core and the tip of Laval nozzel is defined as the potential core length of coherent jet.

As shown in Fig. 4a, the potential core length with 10 %-CH_4_, 9 %-CH_4_, 8 %-CH_4_, 7 %-CH_4_, 6 %-CH_4_ and 5 %-CH_4_ is 29D_e_, 27D_e_, 25D_e_, 23D_e_, 21D_e_ and 18D_e_, respectively, at room ambient temperature. Under high ambient temperature, the potential core length with 10 %-CH_4_, 9 %-CH_4_, 8 %-CH_4_, 7 %-CH_4_, 6 %-CH_4_ and 5 %-CH_4_ is 34D_e_, 32D_e_, 30D_e_, 28D_e_, 26D_e_ and 24D_e_, respectively, as depicted in Fig. 4b. As previous studies (Mathur 1999b), the higher ambient temperature can prolong potential core of coherent jet, and the more CH_4_-rate also could increase the potential core referring the results.

When the ambient temperature being 1700 K, the length of potential core with 10 %-CH_4_, 9 %-CH_4_, 8 %-CH_4_, 7 %-CH_4_, 6 %-CH_4_ and 5 %-CH_4_ is 1.17, 1.18, 1.20, 1.22, 1.24 and 1.33 times larger than that of the coherent jet at ambient temperature being 298 K, respectively. Therefore, higher ambient temperature would prolong the potential core length, but the increasing trend decelerates with shrouding rate increasing.

Based on the results, the average potential core length of the coherent jet is 3.5 times larger than that of the conventional jet. That means the CH_4_ flow rate could effectively improve the potential core length of the main oxygen jet, which protect the stirring ability of the supersonic oxygen jet.

**Fig. 5:**
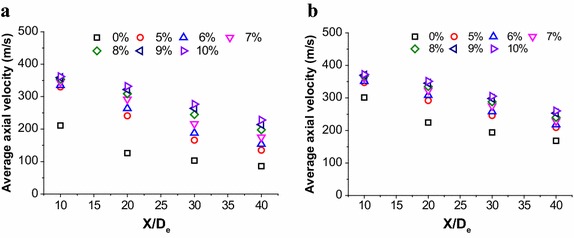
Average axial velocity distributions of supersonic jet at the jet centerline. **a** The coherent jet under room ambient temperature. **b** The coherent jet under high ambient temperature

There are four cross sections have been selected at the jet centerline which radius is 100 mm, and the location is 10D_e_, 20D_e_, 30D_e_ and 40D_e_, respectively. The Fig. 5 shows the average axial velocities of different sections, which are calculated by numerical simulation. Although the coherent jet have a potential core at centerline, the average velocity shows a reduce trend, because periphery of main oxygen would mix with the combustion flame, which suppresses the average velocity of oxygen jet.

The average velocity of the coherent jet at high temperature condition with 10 %-CH_4_, 9 %-CH_4_, 8 %-CH_4_, 7 %-CH_4_, 6 %-CH_4_ and 5 %-CH_4_ is 1.07, 1.09, 1.11, 1.16, 1.21 and 1.25 times higher than that of the coherent jet at room ambient temperature, respectively. Therefore, if the ambient temperature rises, the increasing trend of average velocity is suppressed with CH_4_ flow rate improving, which is the same as increasing trend of potential core.

In this research, the distance between the coherent jet tip and molten bath surface is approximately 20D_e_. When the location of cross section is 20D_e_ at centerline, the average velocity of the coherent jet at high temperature condition with 9 %-CH_4_, 8 %-CH_4_, 7 %-CH_4_, 6 %-CH_4_ and 5 %-CH_4_ is 98.3, 95.4, 92.0, 87.7 and 83.2 % of the 10 %-CH_4_ coherent jet, respectively. It seems that when the CH_4_ flow rate is 9 %-CH_4_ and 8 %-CH_4_, the disparity rate of average velocity is all small than 5 %.

Based on the preview research (Anderson 2013; Alam et al. 2012; Hale 2013), the velocity contour of main oxygen jet would be compressed due to the obstruction of molten bath surface. Therefore, the difference of main oxygen velocity with 10 %-CH_4_, 9 %-CH_4_ and 8 %-CH_4_ may be suppressed at 20D_e_, which makes the coherent jet may achieve the same stirring effect with three kinds of CH_4_ flow rate in EAF steelmaking process.

The Fig. 4 presents numerical simulation results are agreed well with the combustion experiment in this research, and the average error of axial velocity is about 1.7 %. The oxygen is addressed as an ideal-gas in simulation process. However, the oxygen has a deviation from the ideal gas, when the ambient temperature is improving, referring to the previous study (Irvin and Richard 2008). Therefore, the error would be formed under the influence of shrouding flame temperature.

### Total temperature distribution research

**Fig. 6:**
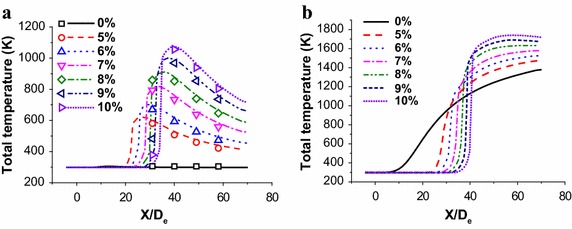
Total temperature distributions of the coherent jet at the jet centerline. **a** The coherent jet under room temperature. **b** The coherent jet under high temperature

As depicted in Fig. 6, the simulations using the EDC model with full detailed chemistry are performed to predict the flame characteristics with various CH_4_ flow rates. In this study, the total temperature distributions at the jet centerline are measured only at cold ambient temperature, and the simulation results agree well with the combustion experiment data.

**Fig. 7:**
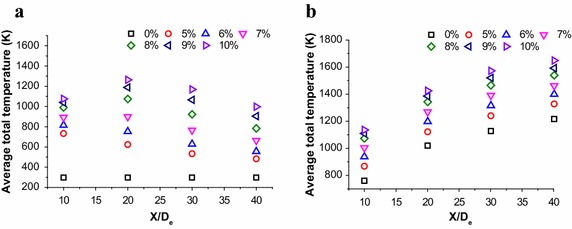
Average total temperature distributions of supersonic jet at the jet centerline. **a** The coherent jet under room ambient temperature. **b** The coherent jet under high ambient temperature

When oxygen jet passes though the Laval nozzle, the pressure potential energy is transformed into the kinetic energy. During this process, although the energy form of oxygen jet is changing, the total energy of oxygen remains unchanged, and then the central jet mixes with combustion flame at the end of the potential core. With a great temperature gradient, the flame transmits thermal energy into oxygen jet, which makes centre of oxygen jet rise rapidly. At last, both kinetic energy and thermal energy of oxygen jet keeps reducing due to the energy gradient between the jet and the ambient flow. As a result, total temperature of the jet gradually approaches the ambient temperature. As described above, the total temperature at jet centerline just one of flow field characteristics, which could not represent the total stirring ability of coherent jet. The average total temperatures of different sections are studied, as shown in Fig. 7.

Under room ambient temperature, when the CH_4_ flow rate is small than 6 %-CH_4_, the average total temperature show a reduce trend with X/D_e_ increasing. However, when the CH_4_ flow rate is bigger than 7 %-CH_4_, the average total temperature increases first, and then approaches to the ambient temperature at centerline direction, as presented in Fig. 7a.

**Fig. 8:**
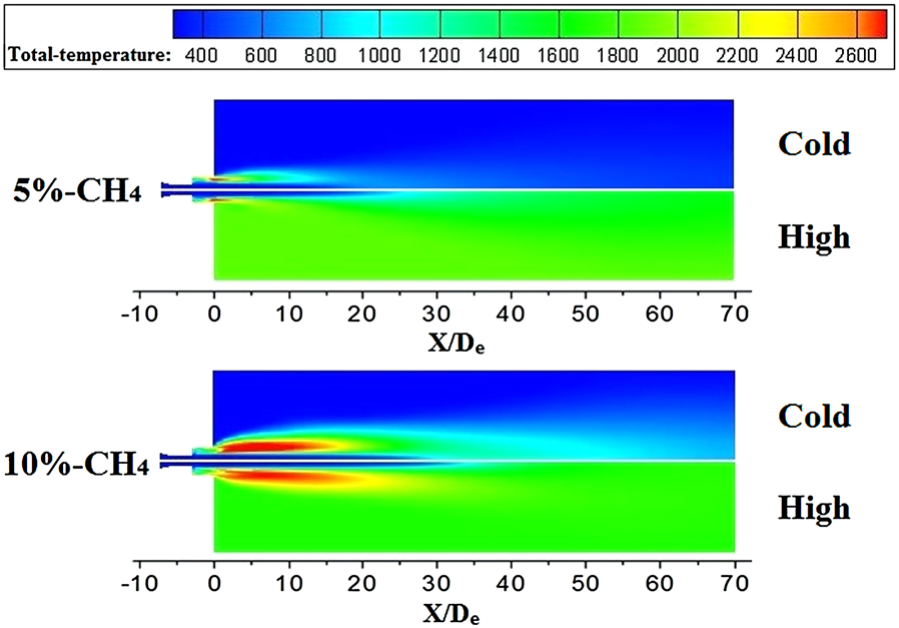
Total temperature distributions of the coherent jet on longitudinal section at cold and high ambient temperature

It seems that the high-temperature zone (the total temperature >2600 K) is formed near the exit of coherent, when CH_4_ flow rate is small, as depicted in Fig. 8. With the combustion flame flowing to the downstream, the thermal energy of coherent jet transmits into the ambient gas due to the temperature gradient, which makes its average total temperature keep reducing. When CH_4_ flow rate is bigger, the high-temperature zone is enlarged making the average total temperature of combustion flame higher at 20X/D_e_. At the same time, the average total temperature of main oxygen increase, because the peripheral of oxygen jet absorbs heat from flame. As a result, the average total temperature at 20X/D_e_ is higher than that at 10X/D_e_. And then, the total temperature reduce rate of flame is bigger than total temperature rise rate of oxygen jet, which makes the average total temperature of coherent jet decreases with X/De increasing.

When the ambient temperature is high, the average total temperature shows an increasing trend with different CH_4_ flow rates, as presented in Fig. 7b. Although the CH_4_ has been exhausted, the combustion flame could keep its total temperature being 1700 K, because of the high ambient temperature. In the meantime, the main oxygen jet could keep absorbing heat until its total temperature being 1700 K. Therefore, on the cross sections, the average total temperature of coherent jet increases, because of the limited reduction rate of flame total temperature and increasing trend of oxygen jet total temperature.

The average total temperature of the coherent jet at high temperature condition with 10 %-CH_4_, 9 %-CH_4_, 8 %-CH_4_, 7 %-CH_4_, 6 %-CH_4_ and 5 %-CH_4_ is 1.28, 1.33, 1.43, 1.58, 1.76 and 1.92 times higher than that of the coherent jet at room ambient temperature, respectively. Therefore, if the ambient temperature rises, the increasing trend of average total temperature is suppressed with CH_4_ flow rate improving, which is the same as increasing trend of average velocity.

When the location of cross section is 20D_e_ at centerline, the average total temperature of the coherent jet at high temperature condition with 9 %-CH_4_, 8 %-CH_4_, 7 %-CH_4_, 6 %-CH_4_ and 5 %-CH_4_ is 97.3, 94.2, 89.1, 84.1 and 78.8 % of the 10 %-CH_4_ coherent jet, respectively.

It seems that when the CH_4_ flow rate is 9 %-CH_4_ and 8 %-CH_4_, the disparity rate of average velocity is all small than 6 %. Therefore, the 9 %-CH_4_ and 8 %-CH_4_ may achieve the same stirring effect in EAF steelmaking process.

### Industrial application research

Based on the combustion experiment and numerical simulation study, the 10 %-CH_4_, 9 %-CH_4_ and 8 %-CH_4_ may achieve the same stirring effect in a 75t electrical arc furnace. In order to study metallurgical effects and technical indicators of the various CH_4_ flow rate in steelmaking process, 10 %-CH_4_, 8 %-CH_4_ and 6 %-CH_4_ are adopted in a 75 t electrical arc furnace. There are 180 heats collected in the industrial smelting process, and each CH_4_ flow rate has same heats. Molten steel component, steelmaking time and dephosphorization rate are analyzed in this research.

The conditions of liquid iron (prior to steelmaking process), and molten steel (after steelmaking process) are shown in Table 3 along with the average components, temperature, and smelting time. When smelting with different CH_4_ flow rates, the conditions of liquid iron are fundamentally the same, which mean the initial conditions have no influence to the industrial application research.

**Table 3:** Average values analysis of liquid iron and molten steel

Label	Liquid iron			Molten steel				Steelmaking time (min)
	C (%)	P (%)	Temperature (°C)	C (%)	P (%)	[C]·[O] (10^−4^)	Temperature (°C)	
10 %-CH_4_	3.6	0.129	1280	0.078	0.007	0.0053	1605	50.3
8 %-CH_4_	3.6	0.130	1279	0.079	0.007	0.0052	1606	50.4
6 %-CH_4_	3.6	0.129	1280	0.081	0.010	0.0061	1605	54.1

Based on the carbon content and temperature of molten steel are same, both 10 %-CH_4_ and 8 %-CH_4_ make no difference on [C]·[O] and steelmaking time. When the CH_4_ flow rate is 6 %-CH_4_, the average [C]·[O] is improved by 16.2 % and the steelmaking time is increased by 7.4 %. This fact can prove that the stirring ability of coherent jet with 10 %-CH_4_ and 8 %-CH_4_ is fundamentally same. Therefore, the dynamic condition of molten bath remains unchanged, when the CH_4_ flow rate is reduced at appropriate situation as observed by the combustion experiment and numerical simulation.

**Fig. 9:**
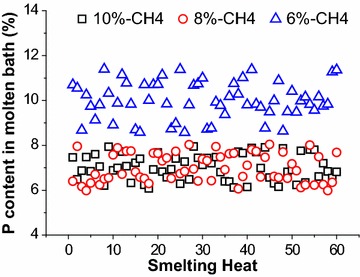
Distribution of phosphor content in molten steel

Figure 9 shows the distribution of phosphorus in molten steel with different CH_4_ flow rates. As shown in Fig. 9, the content of phosphorus in molten steel with 10 %-CH_4_, 8 %-CH_4_ and 6 %-CH_4_ distributes from 0.006 to 0.008, 0.006 to 0.008 and 0.009 to 0.011 mass%, respectively. Based on the results, the average content of phosphorus in molten steel is basically same when the CH_4_ flow rate is 10 %-CH_4_ and 8 %-CH_4_. However, the average content of phosphorus is increased by 0.003 mass % with 6 %-CH_4_, and the dephosphorization rate is reduced by 2.3 %.

**Fig. 10:**
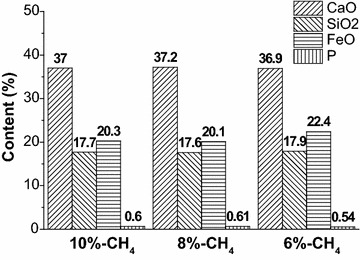
Distribution of CaO, SiO_2_, FeO and P contents in slag

CaO, SiO_2_, FeO and P contents in end-point slag are shown in Fig. 10. While the basicities of slag are same being 2.1, compared with 6 %-CH_4_, average P is increased by 0.07 mass% and FeO is dropped by 2.2 mass% in slag with 10 %-CH_4_ and 8 %-CH_4_. The reduction of FeO loss will be beneficial to improve the metal yield rate.

Based on steelmaking temperature, components of slag layer and molten steel, the equilibrium phosphorus content with various CH_4_ flow rate has been obtained by the Eq. 10 reported by Healy (1970).

10 $$\log \frac{{({\text{mass}}\% \;{\text{P}})}}{{[{\text{mass}}\% \;{\text{P}}]}} = \frac{22,350}{T} + 0.08({\text{mass}}\% \;{\text{CaO}}) + 2.5\log ({\text{mass}}\% \;{\text{FeO}}) - 16$$

The content of equilibrium phosphorus in molten steel is 4.2 × 10^−5^, 4.3 × 10^−5^ and 3.0 × 10^−5^ mass% with 10 %-CH_4_, 8 %-CH_4_ and 6 %-CH_4_, respectively. It is obvious that all equilibrium phosphorus contents are most importantly negligibly small. According to the results of combustion experiment, numerical simulation and industrial application research, it can prove that the reaction rate and stirring effect with 10 %-CH_4_ and 8 %-CH_4_ is same, and both are better than that with 6 %-CH_4_.

Above all, it shows that the stirring ability of coherent jet with 10 %-CH_4_ and 8 %-CH_4_ is fundamental same, and both metallurgical effect is better than with 6 %-CH_4_ in steelmaking process for the 75 t EAF, which agrees well with the results of the combustion experiment and the numerical simulation.

## Conclusions

1. The bigger CH_4_-rate and higher ambient temperature can prolong the potential core of coherent jet, and the numerical simulation results show a good agreement with the combustion experimental data. Moreover, when the ambient temperature rises, the increasing trend of potential core is suppressed with CH_4_ flow rate improving, which is the same as increasing trend of average velocity and total temperature.
2. Based on the results, at high temperature condition, the disparity rate of average velocity and total temperature of the coherent jet, which is between 10 %-CH_4_, 9 %-CH_4_ and 8 %-CH_4_, is all small than 5 and 6 %, respectively. Therefore, the coherent jet may achieve the same stirring effect with three kinds of CH_4_ flow rates in EAF steelmaking process.
3. Compared with 10 %-CH_4_ and 6 %-CH_4_ flow rates at industrial application research, the best CH_4_ flow rate is 8 %-CH_4_, which could stir molten bath well and reduce energy consumption in steelmaking process, with the condition of liquid iron being fundamentally the same.
